# Prevention of guanylyl cyclase–B dephosphorylation rescues achondroplastic dwarfism

**DOI:** 10.1172/jci.insight.147832

**Published:** 2021-05-10

**Authors:** Brandon M. Wagner, Jerid W. Robinson, Yun-Wen Lin, Yi-Ching Lee, Nabil Kaci, Laurence Legeai-Mallet, Lincoln R. Potter

**Affiliations:** 1 Departments of Integrative Biology and Physiology and; 2 Biochemistry, Molecular Biology, and Biophysics, University of Minnesota, Minneapolis, Minnesota, USA.; 3 Institute for Cellular and Organismic Biology, Academia Sinica, Taipei, Taiwan.; 4 Université de Paris, Imagine Institute, Laboratory of Molecular and Physiopathological Bases of OsteochonDrodysplasia, INSERM UMR 1163, F-75015, Paris, France.

**Keywords:** Bone Biology, Bone development, Bone disease, Guanylate cyclase

## Abstract

Activating mutations in the fibroblast growth factor receptor 3 (FGFR3) or inactivating mutations in guanylyl cyclase–B (GC-B), also known as NPR-B or Npr2, cause short-limbed dwarfism. FGFR3 activation causes dephosphorylation and inactivation of GC-B, but the contribution of GC-B dephosphorylation to achondroplasia (ACH) is unknown. GC-B^7E/7E^ mice that express a glutamate-substituted version of GC-B that cannot be inactivated by dephosphorylation were bred with mice expressing FGFR3-G380R, the most common human ACH mutation, to determine if GC-B dephosphorylation is required for ACH. Crossing GC-B^7E/7E^ mice with FGFR3^G380R/G380R^ mice increased naso-anal and long (tibia and femur), but not cranial, bone length twice as much as crossing GC-B^7E/7E^ mice with FGFR3^WT/WT^ mice from 4 to 16 weeks of age. Consistent with increased GC-B activity rescuing ACH, long bones from the GC-B^7E/7E^/FGFR3^G380R/G380R^ mice were not shorter than those from GC-B^WT/WT^/FGFR3^WT/WT^ mice. At 2 weeks of age, male but not female FGFR3^G380R/G380R^ mice had shorter long bones and smaller growth plate hypertrophic zones, whereas female but not male GC-B^7E/7E^ mice had longer bones and larger hypertrophic zones. In 2-week-old males, crossing FGFR3^G380R/G380R^ mice with GC-B^7E/7E^ mice increased long bone length and hypertrophic zone area to levels observed in mice expressing WT versions of both receptors. We conclude that preventing GC-B dephosphorylation rescues reduced axial and appendicular skeleton growth in a mouse model of achondroplasia.

## Introduction

Achondroplasia (ACH) is the most common form of human skeletal dwarfism, which affects about 1 in 20,000 live births worldwide (1–3). ACH is disproportionate dwarfism. Both the appendicular and axial skeleton are affected. Skeletal defects include rhizomelic bowing of long bones, macrocephaly, midface hypoplasia, maxillary hypoplasia, depressed nasal bridges, and spinal foramen magnum stenosis. Adults with ACH have an average height of just over 4 feet, and more than 80% of ACH cases spontaneously occur through mutations in sperm associated with increased paternal age (4). The vast majority of ACH cases result from a single amino acid substitution in fibroblast growth factor receptor 3 (FGFR3) that increases the tyrosine kinase activity of the receptor (2, 3). The most common missense mutation in humans is p.Gly380Arg. Cell culture studies determined that mutations that cause more deleterious ACH phenotypes result in greater increases in phosphorylation and activation of FGFR3 (5). Murine transgenic studies demonstrated that expression of Fgfr3 gain-of-function mutations (p.Gly380Arg) in chondrocytes and osteoblasts results in ACH (6, 7).

FGFR3 is a tyrosine kinase receptor that signals through multiple downstream pathways including: ERK1/2–MAPK, p38–MAPK, PI3K–AKT, STATs, and PLCγ (1). In the growth plate, FGFR3 activates both STATs and the ERK1/2 kinases (8). Although disruption of the Stat1 gene rescued the chondrocyte proliferation deficiency in *Fgfr3^G374R/+^* mice, these mice still developed an ACH-like phenotype (9). In contrast, activated MEK1 expression in chondrocytes of mice lacking active Stat1 yielded an ACH-like phenotype with a decreased hypertrophic chondrocyte zone but no decrease in chondrocyte proliferation (9). Numerous ACH mouse models have been generated with bone and growth plate cartilage anomalies associated with small proliferative and hypertrophic chondrocyte area and less matrix secretion (6, 7). Recently, the mouse *Fgfr3* gene was replaced with the human FGFR3-G380R cDNA to generate a humanized drug discovery model (10). These mice recapitulate the disproportionate shortening of the limbs, kyphosis, and midface hypoplasia observed in ACH patients, but unlike humans and other ACH murine models, expression of 2 FGFR3-G380R alleles (FGFR3^G380R/G380R^) is required for maximum skeletal reductions in these animals (10).

Guanylyl cyclase–B (GC-B) is a membrane enzyme that produces cGMP when activated by C-type natriuretic peptide (CNP) (11–14). Under basal conditions, the pseudokinase domain of GC-B is phosphorylated on multiple serines and threonines, which is required to transmit the CNP activation signal to the catalytic domain (15–17). However, prolonged CNP exposure or brief exposure to antagonistic hormones or growth factors results in GC-B dephosphorylation and inactivation (15, 18–22). Phosphorylation is absolutely required for CNP-dependent activation of GC-B, and alanine substitutions for the known phosphorylation sites yield an enzyme that is not activated by CNP (15–17). In contrast, conversion of the phosphorylated serines and threonines to glutamate to constitutively mimic the negative charge of the phosphorylated residues produces an enzyme that is activated by CNP but not inactivated by dephosphorylation (20–24). GC-B–KO mice or GC-B mice with alanine substitutions for the phosphorylated serines and threonines are dwarfed and have smaller growth plates that are similar to those of ACH mice (17, 25, 26). In humans, inactivating mutations in both alleles of GC-B cause acromesomelic dysplasia Maroteaux–type (AMDM-type) dwarfism (26–28), whereas CNP overexpression or constitutively activating mutations in GC-B cause skeletal overgrowth (29–33). Natriuretic peptide clearance receptor (NPR-C) also binds CNP and degrades it (34). Inactivation of NPR-C (35–37) or elevated blood osteocrin concentrations (38), a natural decoy peptide for NPR-C (39–41), increase both plasma CNP levels and long bone growth (38).

Plasma CNP is elevated in human ACH and AMDM patients (42–44). Importantly, ACH mice overexpressing a CNP transgene (33) — or s.c. injected with CNP (45) or the proteolytically resistant CNP analog, BMN-111 (46) — display increased hypertrophic chondrocytes and matrix production that is correlated with ERK1/2 but not STAT3 inhibition (33, 46). However, activation of p38 MAPK is also associated with CNP/GC-B–dependent increases in endochondral bone growth (47, 48).

Both FGFR3 and GC-B are highly expressed in proliferating chondrocytes, and reciprocal antagonism between the FGF-FGFR3 and CNP–GC-B pathways has been reported by multiple investigators (33, 45, 49, 50). Specifically, CNP–GC-B signaling was shown to inhibit FGFR3-dependent MAPK activation via protein kinase G regulation of Raf-1 (49, 51). Conversely, FGF inhibits CNP-dependent cGMP elevations by increasing GC-B dephosphorylation (24, 52). Importantly, FGF failed to inactivate GC-B–7E in cell culture and in live tibial tissue, consistent with FGF-dependent inactivation of GC-B requiring decreased receptor phosphorylation (24, 52). Since inactivation of GC-B causes a similar phenotype to that observed with FGFR3 gain-of-function mutations, and FGFR3 activation causes GC-B dephosphorylation and inactivation, we hypothesized that GC-B dephosphorylation is required for the FGFR3-dependent ACH. If this hypothesis is correct, then preventing GC-B dephosphorylation by substituting glutamates for known phosphorylation sites should prevent or reduce the severity of FGFR3-dependent ACH.

To test this hypothesis, we crossed mice where 1 allele of the FGFR3 gene was replaced with the human FGFR3-G380R gain-of-function gene (10) with mice expressing one GC-B–7E allele (22) to generate mouse lines of all possible genetic combinations. We observed that both naso-anal and long bone length of the FGFR3^G380R/G380R^ GC-B^7E/7E^ mice was greater than that for FGFR3^G380R/G380R^ GC-B^WT/WT^ mice. Importantly, we also observed that the hypertrophic zones of 2-week-old growth plates of the FGFR3^G380R/G380R^ GC-B^7E/7E^ male mice were the same size as those in WT male mice. Thus, prevention of GC-B dephosphorylation at an early age improved defective chondrocyte differentiation sufficiently to allow normal long bone growth.

## Results

### Generation of the mouse lines.

**Figure 1:**
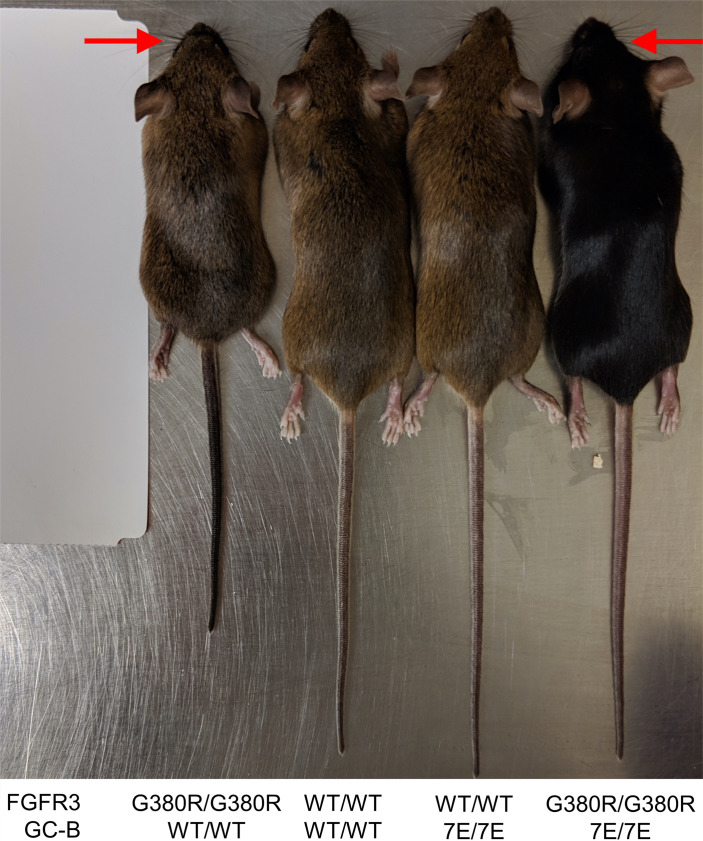
Expression of 2 GC-B^7E/7E^ alleles rescues the naso-anal length but not the midface hypoplasia defect in FGFR3^G380R/G380R^ mice. Male 16-week-old mice of the indicated genotype are shown for visual comparison. The WT or mutant versions of the FGFR3 or GC-B genes that are expressed in each line are shown at the bottom of the figure. The red arrows indicate midface hypoplasia.

**Figure 2:**
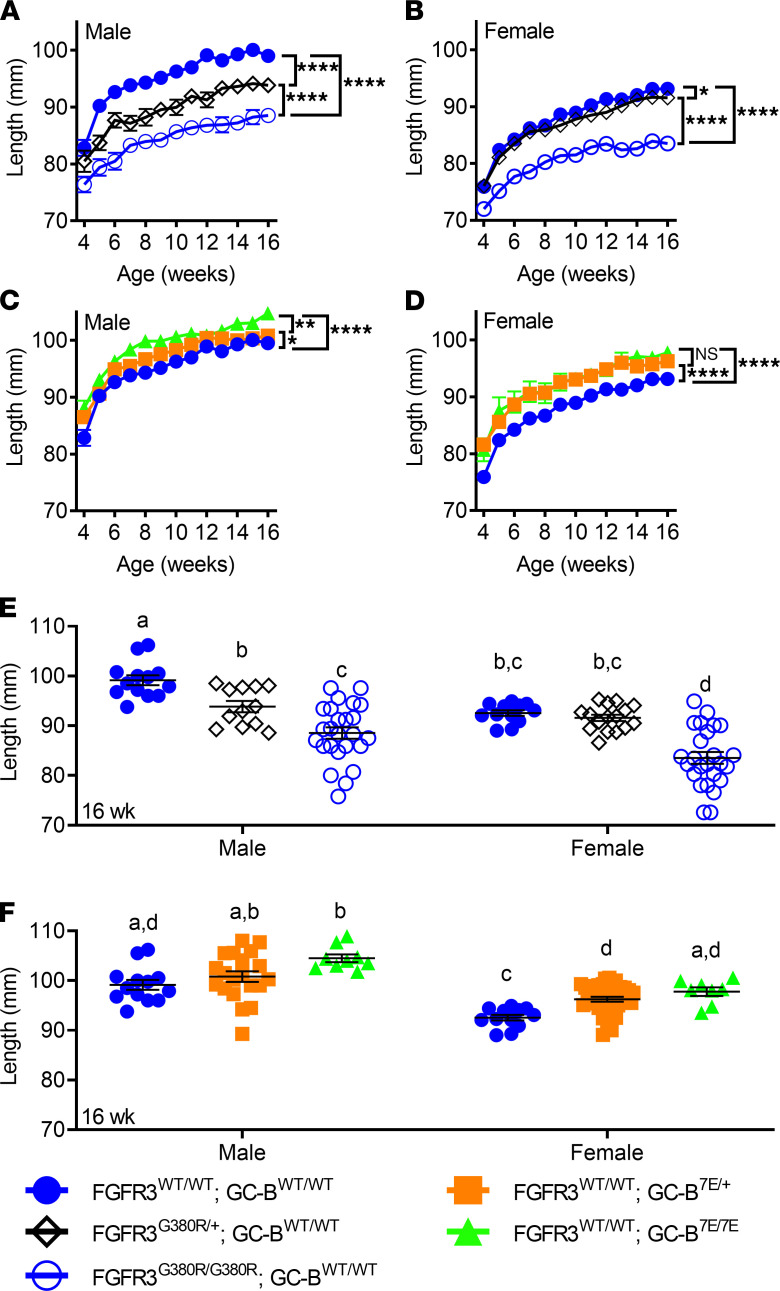
Naso-anal lengths of FGFR3^WT/WT^, FGFR3^G380R/+^, FGFR3^G380R/G380R^, GC-B^WT/WT^, GC-B^7E/+^, or GC-B^7E/7E^ mice according to sex. (**A** and **B**) Comparison of naso-anal lengths with zero, 1, or 2 alleles of FGFR3-G380R in a WT-GC-B background in male (**A**) or female (**B**) mice. (**C** and **D**) Comparison of zero, 1, or 2 alleles of GC-B–7E in a WT-FGFR3 background in male (**C**) or female (**D**) mice. (**E**) Comparison of naso-anal lengths with zero, 1, or 2 alleles of FGFR3-G380R in a WT–GC-B background in male and female mice at 16 weeks of age. (**F**) Comparison of zero, 1, or 2 alleles of GC-B–7E in a WT-FGFR3 background in male and female mice at 16 weeks of age. *, **, and **** indicate statistical significance at *P* < 0.05, 0.01, and 0.0001, respectively. *n* = 12–35 male and 6–46 female mice for each data point. For **E** and **F**, treatments with different letters are significantly different from one another where *P* < 0.05

To evaluate the contribution of GC-B dephosphorylation to ACH, we crossed the GC-B^7E/+^ mice as originally described as Npr^7E/+^ by Shuhaibar et al. (22) or as GC-B^7E/+^ by Robinson et al. (53) with the FGFR3^G380R/+^ mice originally described as FGFR3^ACH/+^ by Lee et al. (10) to generate all the murine lines shown in Figure 1 and Figure 2. The F1 cross-generated F2 littermates of every possible combination of the FGFR3 and GC-B genes in a mixed C57BL/6 and 129 Sv background. The F2 pups were weaned and genotyped 21 days after birth. Naso-anal measurements were performed once weekly from 4 to 16 weeks of age. Initial visual characterization of 16-week-old male mice indicated that expression of 2 GC-B–7E alleles rescued the majority of the naso-anal length defect but failed to rescue the midface hypoplasia defect in mice expressing 2 FGFR3^G380R^ alleles that was obvious to the naked eye (Figure 1).

### FGFR3-G380R and GC-B–7E mice exhibit sex-specific gene-dosage effects.

Although the effects of FGFR3-G380R and GC-B–7E expression on naso-anal length of mixed male and female populations have been described (10, 24), the effects of sex have not been reported for either murine receptor. Using repeated measures analysis from 4 to 16 weeks of age, we observed that expression of 1 allele of FGFR3-G380R decreased skeletal length in male mice by 4.7% but only decreased skeletal length by 1.3% in female mice compared with WT controls (Figure 2, A and B). Expression of 2 FGFR3-G380R alleles further decreased skeletal length in both sexes, but bone length was decreased to a greater extent (11.4%) in males than in females (8.6%) (Figure 2, A and B). In contrast, expression of a single allele of GC-B–7E increased skeletal length 3.7% in females but only increased skeletal length 1.8% in males (Figure 2, C and D). Expression of 2 alleles of GC-B–7E increased skeletal length 4.1% in males but increased skeletal length 5.3% in females (Figure 2, C and D).

When comparing naso-anal length differences at a single age of 16 weeks, single and double FGFR3-G380R alleles decreased naso-anal length of male mice 5.7% and 11.0%, but females were reduced by 1.7% and 10.3%, respectively (Figure 2E). In contrast, single and double alleles of GC-B–7E increased naso-anal length 1.7% and 5.4% in males and 3.3% and 5.0% in females, respectively (Figure 2F). Thus, a single allele of FGFR3-G380R produces a significant decrease in long bone length in adult male but not female mice. Conversely, a single allele of GC-B–7E significantly increased long bone growth in females but not males.

### GC-B^7E/7E^ expression increases naso-anal length more in ACH mice than in WT mice.

**Figure 3:**
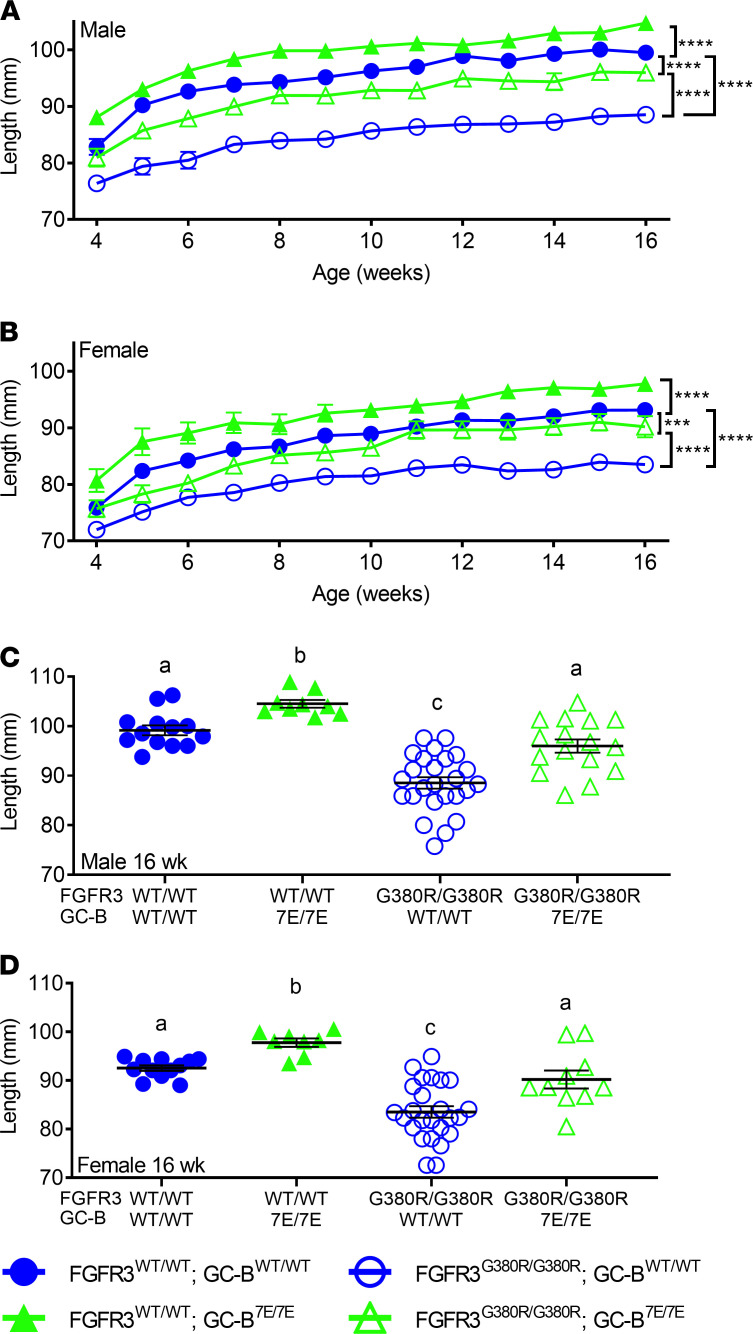
Expression of 2 GC-B–7E alleles rescues FGFR3-G380R–dependent naso-anal length reductions. (**A** and **B**) Comparison of naso-anal lengths in male (**A**) or female (**B**) mice with 2 alleles of GC-B–7E in a homozygous WT-FGFR3 or FGFR3-G380R background from 4 to 16 weeks of age. (**C** and **D**) Comparison of 16-week-old naso-anal lengths in male (**C**) or female (**D**) mice expressing 2 alleles of GC-B–7E in a WT-FGFR3 or homozygous FGFR3-G380R background. *** and **** indicate statistical significance at *P* < 0.001 and 0.0001, respectively, where *n* = 7–35 for male and 4–46 female mice for each data point for **A** and **B**. For **C** and **D**, treatments with different letters are significantly different from one another, where *P* < 0.05 and *n* = 8–25.

To evaluate the contribution of GC-B dephosphorylation to ACH, we generated the mouse lines shown in Figure 3, A and B. These data indicate that expressing 2 alleles of GC-B–7E in mice with 2 alleles of FGFR3-G380R increases naso-anal length to a greater extent than expressing 2 alleles of GC-B–7E in mice with 2 WT FGFR3 alleles. Specifically, GC-B^7E/7E^ expression in an ACH background increased naso-anal length 8.4% and 6.7% in male and female mice, respectively, compared with 4.1% and 5.3% increases, respectively, in FGFR3^WT/WT^ mice. When examining naso-anal growth at a single time period of 16 weeks, expression of 2 GC-B–7E alleles increased growth by 5.3% in males and 4.7% in females in FGFR3^WT/WT^ mice but by 8.4% and 8.0%, respectively, in FGFR3^G380R/G380R^ mice (Figure 3, C and D). The fact that GC-B–7E expression increases naso-anal length more in FGFR3^G380R/G380R^ mice than in FGFR3^WT/WT^ mice is consistent with GC-B dephosphorylation contributing to the reductions in naso-anal growth associated with ACH. In other words, GC-B–7E prevents FGFR3^G380R/G380R^–dependent reductions in the axial skeleton.

### GC-B–7E increases individual long bone length more in ACH mice than in WT mice.

**Figure 4:**
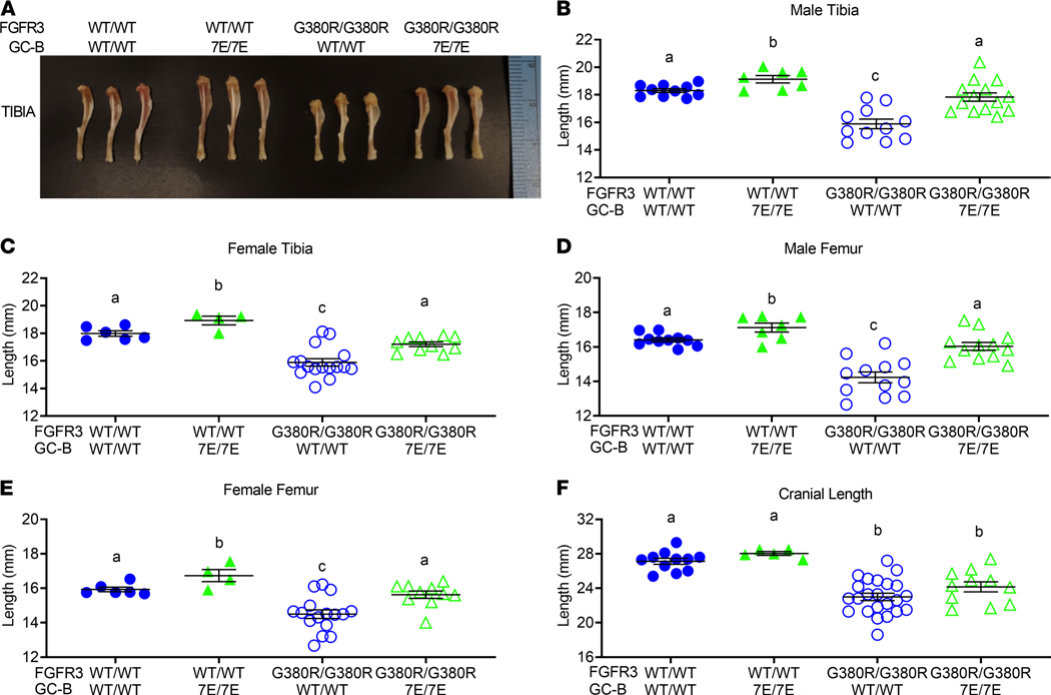
GC-B^7E/7E^ expression rescues long bone but not cranial length abnormalities of ACH in 16-week-old mice. Femurs, tibias, and craniums from 16-week-old male and female GC-B^WT/WT^ FGFR3^WT/WT^, GC-B^7E/7E^ FGFR3^WT/WT^, GC-B^WT/WT^ FGFR3^G380R/G380R^, and GC-B^7E/7E^ FGFR3^G380R/G380R^ mice were excised and measured. (**A**) Representative tibias from 16-week-old male mice of the indicated genotypes. (**B**–**F**)Lengths of male tibias (**B**), female tibias (**C**), male femurs (**D**), female femurs (**E**), and mixed male and female craniums (**F**). *n* = 4–23 mice per genotype. Treatments with different letters are significantly different from one another, where *P* < 0.05.

Femurs and tibias from male and female GC-B^WT/WT^ FGFR3^WT/WT^, GC-B^7E/7E^ FGFR3^WT/WT^, GC-B^WT/WT^ FGFR3^G380R/G380R^, and GC-B^7E/7E^ FGFR3^G380R/G380R^ mice were excised and measured at 16 weeks of age. Homozygous GC-B–7E expression in either the ACH (FGFR3^G380R/G380R^) or WT (FGFR3^WT/WT^) background resulted in greater long bone growth (Figure 4). However, the 12.6% and 7.9% increase in femur length observed for male and female GC-B^7E/7E^ mice expressing 2 FGFR3-G380R alleles was significantly greater than the 4.3% and 5.0% increase in femur length observed in male and female GC-B^7E/7E^ mice expressing 2 WT FGFR3 alleles (Figure 4, D and E). In fact, expression of 2 GC-B–7E alleles restored femoral and tibial lengths in both male and female FGFR3^G380R/G380R^ mice to lengths that were not significantly different from values observed for mice expressing 2 WT alleles for both receptors (Figure 4, B and C). These data indicate that GC-B–7E expression prevents FGFR3^G380R/G380R^–dependent reductions in long bone length and improves the appendicular skeleton.

### Cranial length and midface hypoplasia are not rescued by GC-B–7E expression.

Although cranial length was slightly longer in mixed populations of male and female GC-B^7E/7E^ mice compared with mixed populations of GC-B^WT/WT^ mice, and the cranial length of the FGFR3^G380R/G380R^ mice with 2 GC-B–7E alleles (24.15 mm) was slightly greater than that for mice with 2 GC-B-WT alleles (23.00 mm) (*P* = 0.15) (Figure 4F), post hoc power calculations indicated that our study was significantly underpowered for this analysis (power, 36%). Based on the differences observed in our initial study, we would need 50 mice per group to reach 80% power, which is prohibitive. Consistent with the lack of differences in cranial length data, GC-B^7E/7E^ expression failed to rescue the midface hypoplasia and macrocephaly exhibited by the FGFR3^G380R/G380R^ mice in Figure 1.

### FGFR3-G380R and GC-B–7E expression affect long bone growth in 2-week-old mice a in sex-specific manner.

**Figure 5:**
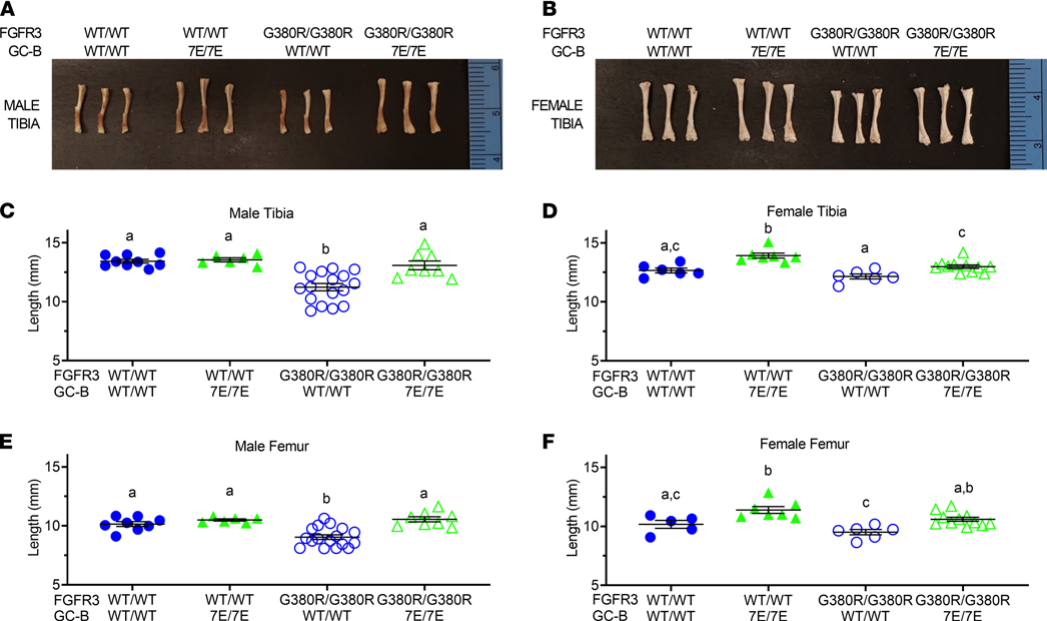
GC-B^7E/7E^ expression rescues FGFR3^G380R/G380R^–dependent shortening of 2-week-old male tibias and femurs. Femurs and tibias from 2-week-old male and female GC-B^WT/WT^ FGFR3^WT/WT^, GC-B^7E/7E^ FGFR3^WT/WT^, GC-B^WT/WT^ FGFR3^G380R/G380R^, and GC-B^7E/7E^ FGFR3^G380R/G380R^ mice were excised and measured. (**A** and **B**) Photographs of representative tibias from 2-week-old male (**A**) or female (**B**) mice of the indicated genotypes. (**C**–**F**) Lengths of male tibias (**C**), female tibias (**D**), male femurs (**E**), and female femurs (**F**). *n* = 5–13 mice per genotype. Treatments with different letters are significantly different from one another, where *P* < 0.05.

To better define the earliest time at which the effects of the FGFR3-G380R and GC-B–7E alleles affect long bone growth, we measured tibia and femur length from 2-week-old male and female FGFR3^WT/WT^ GC-B^WT/WT^, FGFR3^WT/WT^ GC-B^7E/7E^, FGFR3^G380R/G380R^ GC-B^WT/WT^, and FGFR3^G380R/G380R^ GC-B^7E/7E^ mice (Figure 5). Interestingly, we observed that tibias and femurs from 2-week-old mice expressing 2 FGFR3-G380R alleles were only shorter in male mice. Conversely, tibias and femurs were only longer in female 2-week-old mice expressing 2 GC-B–7E alleles. As with the older mice, GC-B–7E expression rescued tibia and femur reductions observed in the FGFR3^G380R/G380R^ 2-week-old male mice. Hence, the single gene effects of FGFR3-G380R and GC-B–7E on tibia and femur length occur as early as 2 weeks of age but only in a sex-specific manner. Importantly, GC-B–7E expression rescued the long bone reductions observed for the 2-week-old FGFR3^G380R/G380R^ male mice, as it did for 16-week-old male and female mice.

### Prevention of GC-B dephosphorylation rescues reduced hypertrophic zone area in 2-week-old FGFR3^G380R/G380R^ male mice.

Growth plates from 2-week-old femurs were sectioned, stained, and analyzed as previously described (10). The growth plates from the FGFR3^WT/WT^ GC-B^WT/WT^ mice were compared with growth plates from FGFR3^WT/WT^ GC-B^7E/7E^, FGFR3^G380R/G380R^ GC-B^WT/WT^, and FGFR3^G380R/G380R^ GC-B^7E/7E^ mice to determine how increased GC-B activity affects hypertrophic zone area in mice expressing mutant and WT FGFR3. Specifically, we examined if the expression of GC-B–7E increased hypertrophic zone area to a greater extent in FGFR3^G380R/G380R^ growth plates compared with FGFR3^WT/WT^ growth plates. Previous murine studies indicated that CNP primarily increases chondrocyte hypertrophy (differentiation), as opposed to proliferation (54).

**Figure 6:**
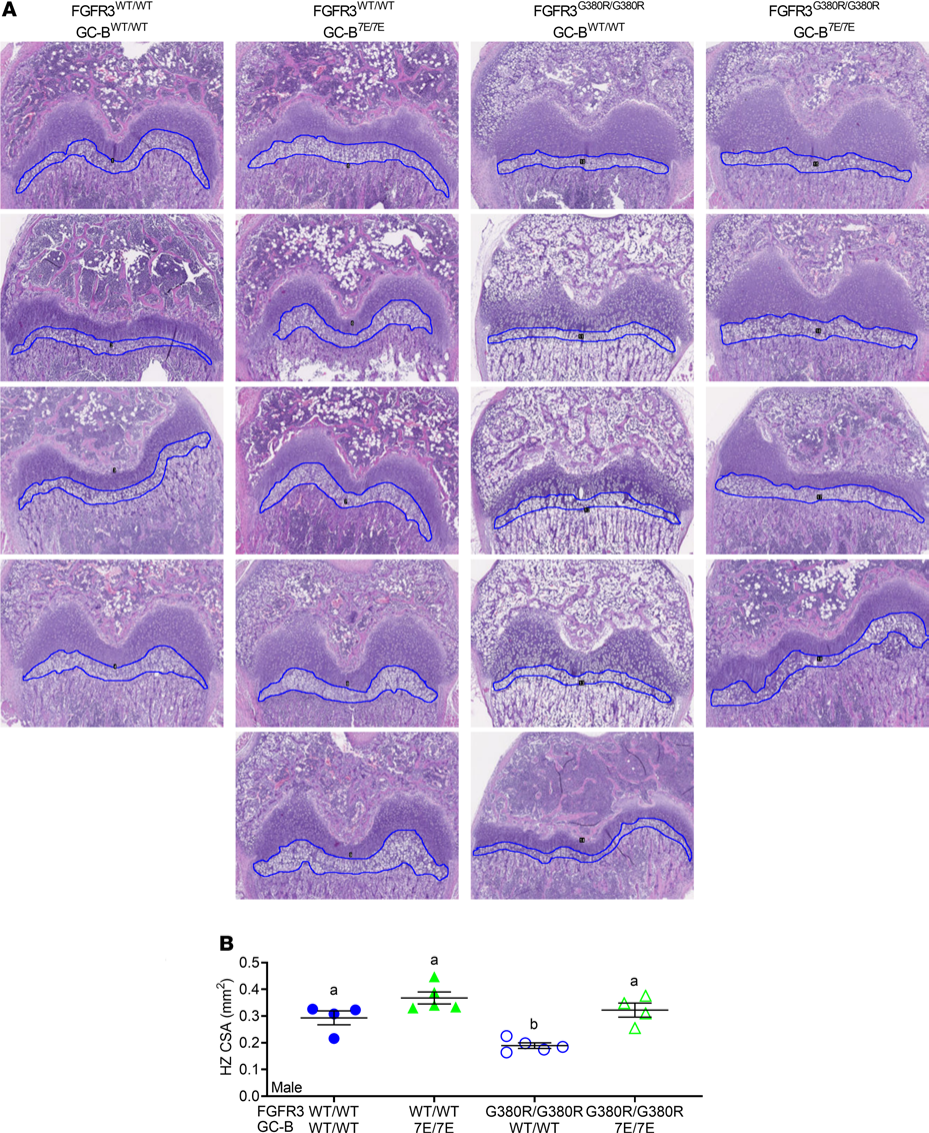
GC-B^7E/7E^ expression rescues growth plate hypertrophic zone reductions in FGFR3^G380R/G380R^ male mice. Femoral growth plates from 2-week-old male mice were sectioned and stained with H&E, and the hypertrophic zone (HZ) cross-sectional area (CSA) was measured. (**A**) Images at 5× magnification were used for HZ CSA. Blue outlines indicate HZ CSA measured. (**B**) Quantitative graph of HZ CSA from **A**. *n* = 4–5 mice per genotype. Treatments with different letters are significantly different from one another, where *P* < 0.05.

Analysis of growth plates from male mice of each genotype indicated that GC-B^7E/7E^ expression increased the hypertrophic zone area in animals expressing either FGFR3^WT/WT^ or FGFR3^G380R/G380^ (Figure 6). However, the GC-B^7E/7E^–dependent increase in hypertrophic zone area was greater in the FGFR3^G380R/G380R^ mice (70.2% increase, *P* = 0.003) compared with the FGFR3^WT/WT^ mice (29.6% increase, *P* = 0.11). In fact, the hypertrophic zones were not significantly different between the FGFR3^G380R/G380R^ GC-B^7E/7E^ mice and the FGFR3^WT/WT^ GC-B^WT/WT^ mice (*P* = 0.80), which is consistent with GC-B^7E/7E^ expression rescuing the hypertrophic zone defect in the FGFR3^G380R/G380R^ mice.

**Figure 7:**
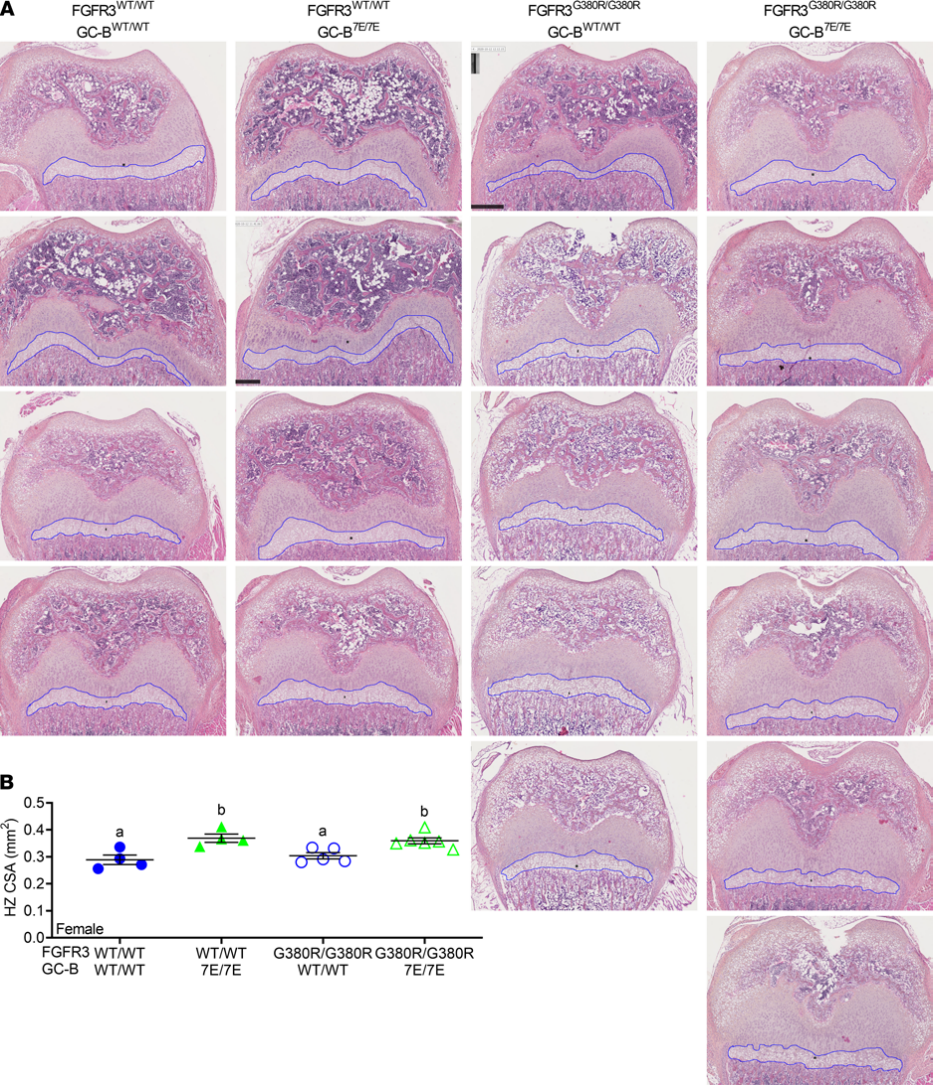
GC-B^7E/7E^ expression expands the hypertrophic zone in FGFR3^WT/WT^ and FGFR3^G380R/G380R^ female mice. Femoral growth plates from 2-week-old female mice were sectioned and stained with H&E, and the hypertrophic (HZ) cross-sectional area (CSA) was measured. (**A**) Images at 5× magnification used for HZ CSA. Blue outlines indicate HZ CSA measured. (**B**) Quantitative graph of HZ CSA from **A**. *n* = 4–6 mice per genotype. Treatments with different letters are significantly different from one another where *P* < 0.05.

In female mice, hypertrophic zone decreases were not observed in FGFR3^G380R/G380R^ compared with FGFR3^WT/WT^ mice at 2 weeks (*P* = 0.87). However, GC-B–7E expression increased hypertrophic zone areas in both FGFR3^WT/WT^ (27.7% increase, *P* = 0.007) and FGFR3^G380R/G380R^ (18.2% increase, *P* = 0.032) female mice (Figure 7, A and B). In contrast to the males, hypertrophic zone area in FGFR3^G380R/G380R^ GC-B^7E/7E^ female mice was significantly larger than that observed in FGFR3^WT/WT^ GC-B^WT/WT^ female mice (*P* = 0.0099), possibly because the FGFR3-G380R mutations failed to decrease long bone growth in the female mice at this early age. Together, these data indicate that the inhibitory effects of the FGFR3-G380R mutations occur earlier in male than female mice, whereas the positive effects of GC-B–7E expression on growth plate area occur earlier in female than male mice.

## Discussion

**Figure 8:**
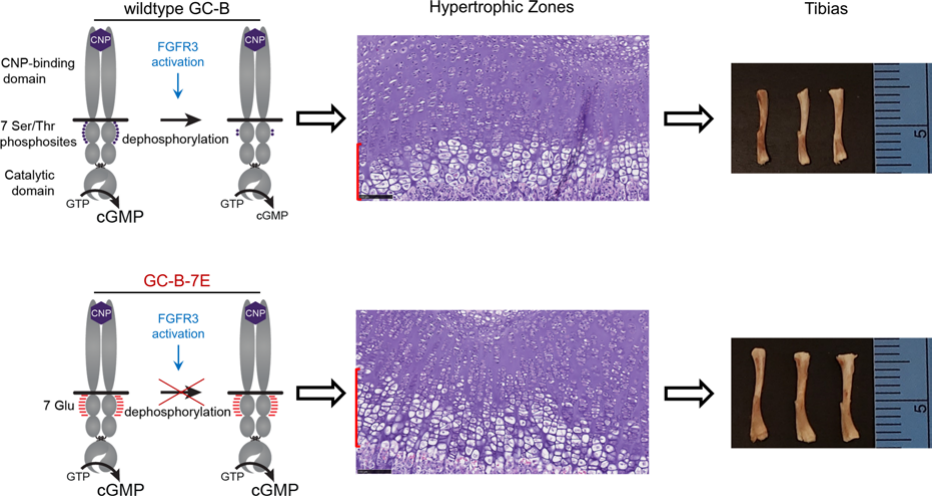
A model depicting how FGFR3-dependent dephosphorylation of GC-B results in achondroplasia. Upper panel: FGFR3 activation increases the dephosphorylation of WT GC-B, which results in reduced chondrocyte cGMP concentrations and smaller growth plate hypertrophic zone area, as depicted by the red bar on the left side of the “Hypertrophic Zones” panel. The reduced hypertrophic zone area ultimately leads to impaired long bone growth that results in shorter tibias. Bottom panel: GC-B–7E cannot be inactivated by dephosphorylation in response to FGFR3 activation, which results in elevated cGMP concentrations, larger hypertrophic zone area, and longer tibias.

In this report, skeletal phenotypes of mice expressing GC-B–7E alleles and FGFR3-G380R alleles were analyzed and described as a function of age. Our results demonstrate that blocking dephosphorylation-dependent inactivation of GC-B rescues the disproportionate dwarfism of the FGFR3^G380R/G380R^ mice by preventing the FGFR3-G380R–dependent decreases in growth plate hypertrophic zone area as graphically summarized in Figure 8. The rescue was observed at multiple biological levels, including naso-anal length and individual femur and tibia length, as well as growth plate hypertrophic zone area. Specific interactions between GC-B and FGFR3 on long bone growth were also identified. For example, crossing the GC-B^7E/7E^ mice with the FGFR3^G380R/G380R^ mice had a greater effect on naso-anal and long bone length than crossing the GC-B^7E/7E^ mice with FGFR3^WT/WT^ mice.

Sex-specific, single allele differences for both genes on long bone growth were also described for the first time, to our knowledge. For single FGFR3-G380R alleles in adult mice, greater skeletal length effects were observed in males, whereas for GC-B–7E, greater effects were observed in females. Increased CNP–GC-B–dependent bone growth in females is consistent with studies showing that CNP expression is more sensitive to exogenous CNP infusion in female compared with male rats (55). Unexpectedly, analysis of 2-week-old femurs and tibias indicated that only males had FGFR3-G380R–dependent reductions in long bone growth, whereas only females exhibited GC-B–7E–dependent increases in bone growth at 2-weeks of age. Histological examination of 2-week-old growth plates confirmed that FGFR3-G380R expression decreased hypertrophic zone area of male but not female growth plates. In contrast, GC-B–7E expression increased the hypertrophic zone area in female but not male growth plates. Importantly, GC-B–7E expression rescued the hypertrophic zone defects in the male FGFR3^G380R/G380R^ mice. In fact, there was no difference in the size of the growth plates from the FGFR3 ^G380R/G380R^/GC-B^7E/7E^ mice compared with mice expressing WT versions of both receptors, which is consistent with a rescue of the reduced hypertrophic zone by GC-B–7E expression. Sex-specific effects of ACH on limb lengthening and bone age delay in children have been described, but whether these effects are related to our results in mice is currently unknown (56, 57).

The differences in naso-anal growth between the various genetic lines changed very little over the 4- to 16-week measurement period, which indicates that the primary effects of GC-B on long bone growth occurs before 4 weeks of age. Large differences in both 2-week-old bones and growth plate hypertrophic zones between mice expressing GC-B–7E and FGFR3-G380R alleles support the idea that the major skeletal effects resulting from the activation of these receptors occurs long before closure of the epiphyseal growth plate. Previously reported increases in murine CNP mRNA at 3 weeks compared with 6 weeks supports the notion that early GC-B activity facilitates endochondral bone growth (38). Identifying when GC-B activation exerts its greatest effect on the growth plate may be important for determining optimal timing of BMN-111 treatment of children with ACH.

While the FGFR3^G380R/G380R^ mice with 2 alleles of GC-B–7E are not dwarfed, they retain the midface hypoplasia that is a hallmark of ACH. This finding suggests that, although GC-B dephosphorylation is a primary mechanism by which the appendicular and axial skeleton is regulated, the majority of the cranial abnormality is not rescued by increased GC-B activity. However, treatment with BMN-111 and the phosphatase inhibitor LB-100 rescued skull base abnormalities in an ex vivo embryonic skull culture assay from Fgfr3^Y367C/+^ mice (58). Thus, a reasonable interpretation of our cranial length data is that, since only a small portion of the skull is stimulated by GC-B, differences in cranial length between GC-B^WT/WT^ and GC-B^7E/7E^ mice are difficult to demonstrate when measuring the total length of the skull. Hence, the majority of the skull growth occurs by membranous ossification, but a small portion at the base of the skull grows by endochondral ossification, which is stimulated by GC-B.

We suggest that GC-B dephosphorylation is both necessary and sufficient for ACH-dependent reductions in long bone growth. Regarding the “sufficiency” claim, a plethora of reports have shown that inactivating mutations in only GC-B result in dwarfism in mice, rats, and humans (17, 26–28, 59–61). Hence, it is clear that inactivation of GC-B, as occurs with dephosphorylation (15, 16), is sufficient to cause ACH-like dwarfism. The data supporting the “necessary” claim are that mice expressing alanine substitutions for the phosphorylated serines and threonines in GC-B are dwarfed (17), and the GC-B^7E/7E^ mice that express a version of GC-B that cannot be inactivated by dephosphorylation are immune to the long bone and growth plate reductions seen in the FGFR3^G380R/G380R^ mice. Thus, if GC-B is never phosphorylated, the mice are dwarfed. Conversely, if GC-B cannot be inactivated by dephosphorylation, as is the case in the GC-B^7E/7E^ mice, then they are resistant to ACH.

Finally, multiple investigators have reported that CNP or BMN-111 (vosoritide) injections increase long bone growth in animal models of ACH (33, 45, 46, 62–66). Recent clinical phase III studies also indicate that vosoritide increases growth curves in achondroplastic children (67–69). The fact that BMN-111 is effective in these various ACH models means that a significant fraction of GC-B is phosphorylated, since dephosphorylated GC-B is not activated by CNP or, presumably, CNP analogs (15, 16). According to our hypothesis, GC-B dephosphorylation resulting from constitutive activation of FGFR3 is a significant part of the molecular explanation for ACH, although we cannot rule out that other FGFR3-activated signaling pathways also participate in this process. Importantly, if preventing GC-B dephosphorylation rescues ACH as we suggest, then therapies that preserve or increase the phosphorylation state of GC-B, should increase long bone growth in ACH models. Consistent with this hypothesis, the phosphatase inhibitor LB-100 was recently shown to synergistically work with BMN-111 to improve long bone growth in mice (58). Future experiments are required to determine if this combination therapy will be safe and effective in other mammalian species, including humans.

## Methods

### Animals.

Male and female WT and GC-B^7E/7E^ mice (originally described as *Npr2^7E/7E^*) were genotyped and maintained as previously described on a C57BL/6J background (22). The FGFR3^G380R/G380R^ mice were genotyped and maintained on a mixed C57BL/6 and 129 Sv line (10). Crosses between the GC-B^7E/+^ C57BL/6J mice and the FGFR^ACH/+^ C57BL/6 and 129 Sv mice produced all the genotypes shown in Figure 1 and Figure 2. Mice were fed standard chow under specific pathogen–free conditions.

### Naso-anal, cranial, tibial, and femoral measurements.

Naso-anal measurements were conducted on conscious male and female mice once weekly from 4 to 16 weeks of age for the duration of the study using a digital caliper. Mice were euthanized at 16 weeks, and their tibias, femurs, and craniums were isolated and measured using a digital caliper. In a separate study, tibia and femur length from 2-week-old mice were determined as described above.

### Growth plate histology.

Two-week-old male and female mice were euthanized; femurs were harvested and fixed in 4% paraformaldehyde for 24 hours and then stored in fresh 4% paraformaldehyde until they were embedded in paraffin and sectioned. The bone sections were then stained with H&E and imaged with a 5× or 10× objective lenses. The upper and lower bounds of the cross-sectional area of the hypertrophic zones were identified by a morphology of columnar, stacked cells that were measured in millimeters in an unbiased manner using ImageJ software (NIH). The freehand selection tool in ImageJ determined the number of square millimeters of the identified hypertrophic region from each section of bone as indicated by the blue outline in the images shown in Figure 6 and Figure 7. In a blinded manner, the square millimeter value from each femoral section was determined and plotted as a single value in Figure 6B and Figure 7B.

### Statistics.

Statistics and graphs were generated with Prism 8 software (GraphPad Software). For the naso-anal growth curves, a mixed-model 2-way ANOVA with repeated measures was applied using a Geisser-Greenhouse correction for sphericity and Tukey’s correction for multiple comparisons. Therefore, the naso-anal length values described under results were derived from repetitive measurements on the same animal at multiple ages. The remaining analyses including the growth plate measurements were performed using a standard, 1-way ANOVA assuming Gaussian distribution and equal SD with Tukey’s correction for multiple comparisons. Except for the naso-anal growth curves, each symbol in a figure represents a measurement from an individual mouse. The longer horizontal bar within each group of symbols represents the mean, and the shorter bars above and below the mean represent the SEM. For the naso-anal growth curves, each point represents the mean length measurement at the indicated age, and bars above and below the mean indicate SEM. Differences were considered significantly different when the *P* value was less than 0.05.

### Study approval.

Animal care was compliant with the University of Minnesota IACUC–approved protocol no. 1806A-35995.

## Author contributions

Study design was contributed by JWR, LLM, and LRP. Naso-anal length measurements were contributed by JWR and BMW. Tibia and femur measurements were contributed by JWR and BMW. Histological data were contributed by NK. Hypertrophic zone measurement was contributed by JWR, BMW, and LLM. Analysis of data was contributed by BMW, JWR, LLM, and LRP. Drafting the manuscript was contributed by JWR, BMW, and LRP. Revising manuscript content was contributed by BMW, JWR, LRP, and LLM. YWL and YCL provided the ACH mouse line, and YCL edited the paper. All authors approved the final version of the manuscript.

## Version 1 03/30/2021

In-Press Preview

## Version 2 05/10/2021

Electronic publication
